# Early Crowdfunding Response to the COVID-19 Pandemic: Cross-sectional Study

**DOI:** 10.2196/25429

**Published:** 2021-02-09

**Authors:** Sameh Nagui Saleh, Christoph U Lehmann, Richard J Medford

**Affiliations:** 1 Department of Internal Medicine University of Texas Southwestern Medical Center Dallas, TX United States; 2 Clinical Informatics Center University of Texas Southwestern Medical Center Dallas, TX United States; 3 Departments of Pediatrics, Bioinformatics, Population & Data Sciences University of Texas Southwestern Medical Center Dallas, TX United States

**Keywords:** crowdfunding, fundraising, GoFundMe, COVID-19, coronavirus, pandemic, natural disasters, disaster relief, fundraise, disaster, cross-sectional, crowdfund, crowdsource, economy, social media, community, distress

## Abstract

**Background:**

As the number of COVID-19 cases increased precipitously in the United States, policy makers and health officials marshalled their pandemic responses. As the economic impacts multiplied, anecdotal reports noted the increased use of web-based crowdfunding to defray these costs.

**Objective:**

We examined the web-based crowdfunding response in the early stage of the COVID-19 pandemic in the United States to understand the incidence of initiation of COVID-19–related campaigns and compare them to non–COVID-19–related campaigns.

**Methods:**

On May 16, 2020, we extracted all available data available on US campaigns that contained narratives and were created between January 1 and May 10, 2020, on GoFundMe. We identified the subset of COVID-19–related campaigns using keywords relevant to the COVID-19 pandemic. We explored the incidence of COVID-19–related campaigns by geography, by category, and over time, and we compared the characteristics of the campaigns to those of non–COVID-19–related campaigns after March 11, when the pandemic was declared. We then used a natural language processing algorithm to cluster campaigns by narrative content using overlapping keywords.

**Results:**

We found that there was a substantial increase in overall GoFundMe web-based crowdfunding campaigns in March, largely attributable to COVID-19–related campaigns. However, as the COVID-19 pandemic persisted and progressed, the number of campaigns per COVID-19 case declined more than tenfold across all states. The states with the earliest disease burden had the fewest campaigns per case, indicating a lack of a case-dependent response. COVID-19–related campaigns raised more money, had a longer narrative description, and were more likely to be shared on Facebook than other campaigns in the study period.

**Conclusions:**

Web-based crowdfunding appears to be a stopgap for only a minority of campaigners. The novelty of an emergency likely impacts both campaign initiation and crowdfunding success, as it reflects the affective response of a community. Crowdfunding activity likely serves as an early signal for emerging needs and societal sentiment for communities in acute distress that could be used by governments and aid organizations to guide disaster relief and policy.

## Introduction

 As the number of COVID-19 cases precipitously accelerated in March throughout the United States, government and public health departments marshalled their pandemic responses. Support efforts quickly became necessary as shortages of medical supplies and testing worsened by the continued spread of the virus affected health care organizations, medical offices, and nursing homes [1]. The first effects of the pandemic were related to health; however, economic ramifications, including rapidly increasing unemployment and loss of revenue, quickly followed the spread of the disease and public health efforts to contain it [2,3]. Temporary and permanent closure of small businesses, furloughing of whole sectors of the economy (eg, airline and restaurant industries), and food price inflation created social, financial, and medical strain on the population [4,5]. Subsequent effects of the pandemic, such as the increased number of deaths and associated funeral costs, added more financial stress to the lives of Americans.

Donation-based web-based crowdfunding has become an increasingly popular tool to finance medical treatment, respond to financial hardships and natural disasters, and defray the downstream economic impacts of personal health care costs [6,7]. Previous research has shown that crowdfunding has been used to address the needs of individuals and communities after natural disasters [8]. However, the crowdfunding success of these donation-based platforms relies more heavily on emotional delivery to engage backers [9] as well as on social media for quick diffusion [10].

Despite anecdotal reports of the increased use of web-based crowdfunding to mitigate the economic impacts of COVID-19 [11], details on how crowdfunding has been used for COVID-19 relief efforts in the United States remain scarce. We evaluated web-based crowdfunding campaigns in the United States in the early stages of the COVID-19 pandemic on the GoFundMe platform, which represents 90% of the social crowdfunding market in the United States as of 2018 [12]. We hypothesized that (1) COVID-19–related campaigns raised more money and were more widely distributed on social media than non–COVID-19–related campaigns during the same timeframe and (2) COVID-19–related campaigns were initiated disproportionately in the early days and weeks of the pandemic, unrelated to the rate of incident COVID-19 cases.

## Methods

### Data Collection and Analysis

On May 16, 2020, we extracted all active US campaigns created between January 1 and May 10, 2020, on GoFundMe, the largest web-based crowdfunding platform in the United States. The time frame reflects approximately two months before and after the World Health Organization declared COVID-19 a pandemic on March 11, 2020. We used the *Beautiful Soup* library [13] to web scrape all data available for the GoFundMe campaigns and excluded all campaigns that originated outside the United States. We list all extracted data fields in Table S1 in Multimedia Appendix 1. We used keywords relevant to the COVID-19 pandemic to identify and label the subset of COVID-19–related campaigns (Table S2 in Multimedia Appendix 1). The University of Texas Southwestern Human Research Protection Program Policies, Procedures, and Guidance did not require institutional review board approval, as all data were publicly available.

We examined weekly new COVID-19–related campaigns juxtaposed with the weekly numbers of new COVID-19 cases both nationally and by state or territory. We evaluated the incidence of campaigns by state adjusted for new COVID-19 cases by week to determine the temporal association between disease spread and campaign initiation. To enable comparison among states, we defined week 0 as the first week in which each state had at least 100 new weekly cases. We displayed all states; however, we only highlighted the top six states by overall campaign per million. Given the presence of zip code–level data, we also explored more granular community trends. We also evaluated changes in COVID-19–related campaigns per million for each state and campaign categories over time. To better understand campaign narratives and motivation, we conducted a natural language processing (NLP) analysis of the campaign text to identify clusters of keywords that represent campaigns [14]. We used the term frequency–inverse document frequency (TF-IDF) technique to create a matrix of all of the words in the campaign narratives weighted by the frequency of the word in that document relative to the frequency of the same word in all documents [15]. We then used k-means clustering on the resulting matrix to identify clusters of campaigns and represent these clusters by the 20 most central words to that cluster [16]. Because there were 19 GoFundMe categories in the COVID-19–related campaign subset (out of 20 possible GoFundMe categories), we chose 19 topic clusters a priori.

We compared campaign characteristics, including fundraising, campaign information, and campaign category, for COVID-19–related and non–COVID-19–related campaigns over the same time period from March 11 to May 10, 2020. We used Mann-Whitney U, chi-square, and Fisher exact tests where appropriate to determine significance. The α level of significance was set a priori at .05, and all significance testing was two-sided. We did not adjust for multiple comparisons, as this was an exploratory study and should be interpreted as hypothesis-generating. Analyses were performed using Python, version 3.7.2 (Python Software Foundation).

### Availability of Data and Materials

The data that support the findings of this study are available upon request. 

## Results

**Figure 1:**
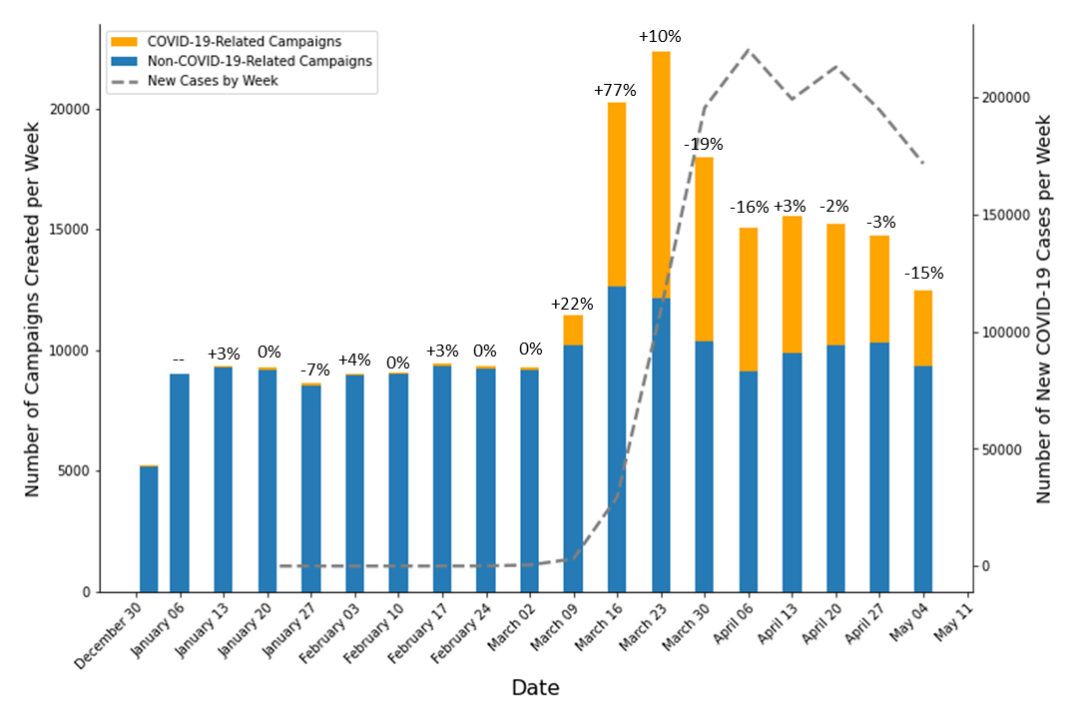
New GoFundMe campaigns by week versus incident COVID-19 cases by week. Percent changes of campaigns created from the previous week are shown above each bar.

Of 310,695 total new campaigns created between January 1 and May 10, 2020, we analyzed 232,827 campaigns from the United States across all categories. Of these campaigns, 51,763/232,827 (22.2%) were identified as COVID-19–related, and these campaigns collectively raised US $237,418,235 by May 10, 2020. The vast majority of COVID-19–related campaigns were established after March 11 (50,828/51,763, 98.2%). New GoFundMe campaigns peaked in mid-March, mainly due to new campaigns related to or referencing COVID-19, and declined in April (Figure 1). Total campaigns increased by 77% and 10% in the weeks of March 16 and 23 and then dipped by 19% and 16% in the following two weeks. In parallel, COVID-19–related campaigns increased by 736% and 512% in the weeks of March 9 and 16 and then eventually decreased by 25% and 23% in the weeks of March 30 and April 6. The COVID-19–related increase in campaigns was observed before the national peak of new COVID-19 cases in April. Subsequently, the incidence of new campaigns declined, while the incidence of new COVID-19 cases remained steady or declined slightly. 

**Figure 2:**
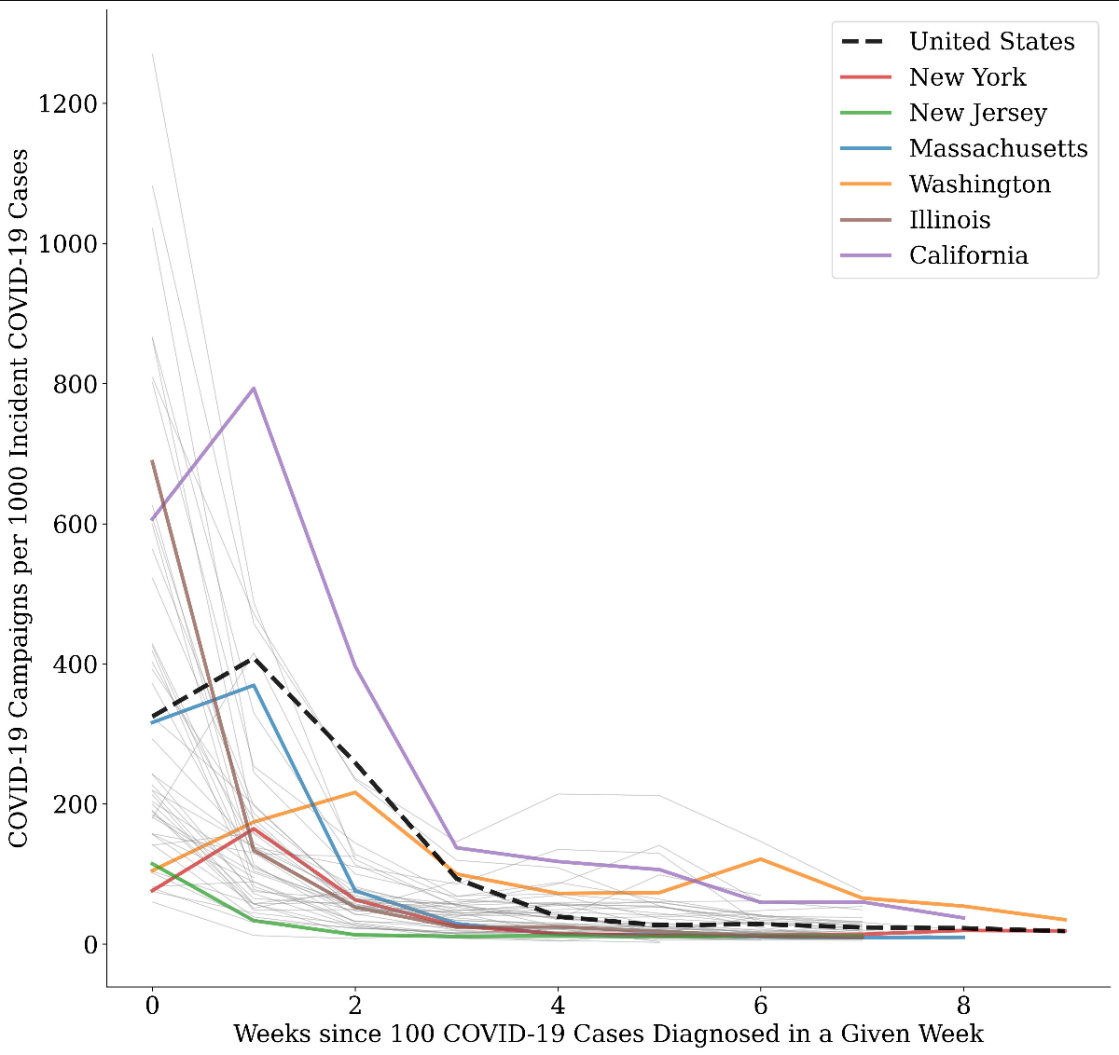
COVID-19–related GoFundMe campaigns per 1000 COVID-19 incident cases for each state. Week 0 is defined as the first week in which a state had more than 100 cases to enable direct comparison among states. All states are shown, but only the top 6 states for total COVID-19 cases per million are highlighted in color (ordered from most to fewest in the legend).

**Figure 3:**
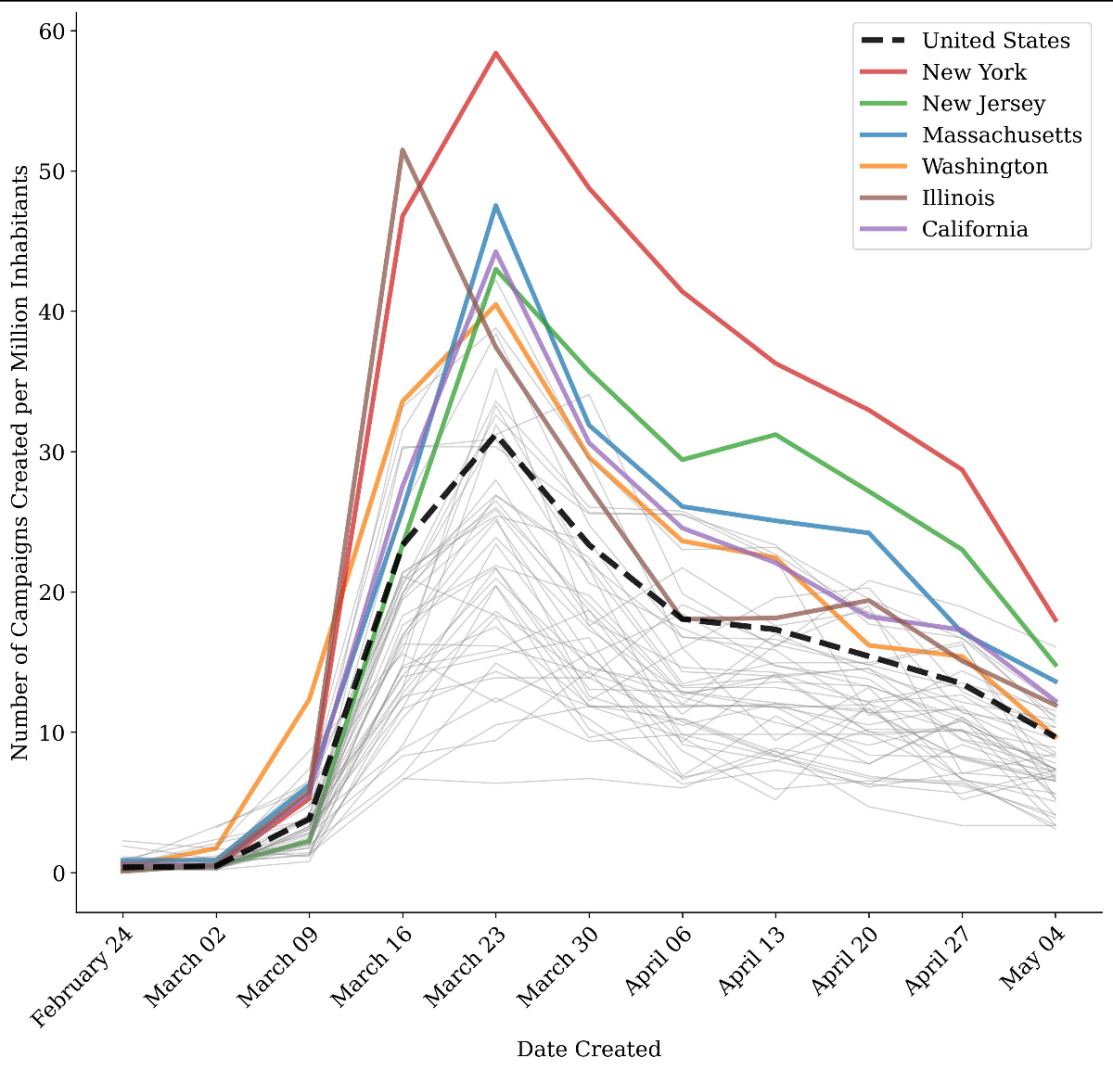
COVID-19–related GoFundMe campaigns per million by week by state. All states are shown, but only the top 6 states for total COVID-19 cases per million are highlighted in color (ordered from most to fewest in the legend). Dates on the x-axis start from February 25, as few COVID-19–related campaigns were present before this date.

Adjusting for the weekly incidence of COVID-19 cases, new COVID-19–related campaigns in all US states peaked within two weeks of the first week when 100 new cases were diagnosed in that state. For many states, the rate of COVID-19–related campaigns per case dropped by at least tenfold after the first two to three weeks (Figure 2). Population-rich states that also experienced early spread of COVID-19, such as New York, New Jersey, Massachusetts, Washington, and Illinois, had the most COVID-19–related campaigns per million inhabitants (Figure 3); however, they were ultimately among the states with the lowest number of COVID-19–related campaigns per 1000 cases during the study period. 

From March 11 to May 10, 2020, non-COVID-19–related campaigns (91,631/142,459, 64.3%) raised a median of US $625 (IQR $135-$2300), while COVID-19–related campaigns (50,828/142,459, 35.7%) raised a median of $930 (IQR $220-$3075), nearly 50% more per campaign (*P*<.001). Fundraising goals were higher in COVID-19–related campaigns (median $5000, IQR $2000-$10,000) than non–COVID-19–related campaigns (median $4000, IQR $1250-$10,000; *P*<.001). Even with the higher fundraising goals, COVID-19–related campaigns (median 25.0%, IQR 5.5%-69.5%) raised a higher percentage of their fundraising goals than non–COVID-19–related campaigns (median 22.2%, IQR 5.0%-65.5%) by date of data extraction (*P*<.001). COVID-19–related campaigns (median 23, IQR 0-163) had significantly more Facebook shares than non–COVID-19–related campaigns (median 0, IQR 0-145; *P*<.001). COVID-19–related campaigns also had longer narratives in their campaign description, were more likely to be listed as a charity, and were more likely to have the campaigner be the same as the beneficiary (Table 1).

**Table 1:** Baseline GoFundMe campaign characteristics stratified by COVID-19–related status. Only campaigns created after March 11, 2020, are included.

Characteristic		Total campaigns (N=142,459)	COVID-19–related campaigns (n=50,828)	Non–COVID-19–related campaigns (n=91,631)	*P* value^a^
**Fundraising**					
	Goal (US $), median (IQR)	5000 (1500-10,000)	5000 (2000-10,000)	4000 (1250-10,000)	<.001
	Raised (US $), median (IQR)	722 (160-2,565)	930 (220-3075)	625 (135-2300)	<.001
	Percent of goal funded (US $), median (IQR)	23.3 (5.2-66.9)	25.0 (5.5-69.5)	22.2 (5.0-65.5)	<.001
	Donors, median (IQR)	12 (3-35)	14 (4-40)	11 (3-32)	<.001
	Amount per donation (US $), median (IQR)	56.3 (35.4-86.8)	59.9 (39.0-92.7)	54.5 (33.8-83.5)	<.001
	Met funding goal, n (%)	20,593 (14.5)	7302 (14.4)	13,291 (14.5)	.48
**Campaign information**					
	Campaigner = beneficiary, n (%)	119,243 (83.7)	43,445 (85.5)	75,798 (82.7)	<.001
	Is listed as a charity, n (%)	7144 (5.0)	3629 (7.1)	3515 (3.8)	<.001
	Words in narrative, median (IQR)	145 (84-240)	187 (115-299)	125 (72-207)	<.001
	Characters in narrative, median (IQR)	56.3 (35.4-86.8)	1072 (652-1729)	691 (393-1154)	<.001
	Active donation days, median (IQR)	23 (14-33)	24 (15-38)	22 (13-31)	<.001
	Facebook shares, median (IQR)	4 (0-152)	23 (0-163)	0 (0-145)	<.001
	GoFundMe hearts, median (IQR)	12 (3-33)	14 (4-38)	10 (3-31)	<.001
**Campaign category (top 8 of 20)**					
	Accidents & Emergencies, n (%)	25,306 (17.8)	10,705 (21.1)	14,601 (15.9)	<.001
	Medical, Illness & Healing, n (%)	22,669 (15.9)	8484 (16.7)	14,185 (15.5)	<.001
	Funerals & Memorials, n (%)	19,577 (13.7)	3242 (6.4)	16,335 (17.8)	<.001
	Community & Neighbors, n (%)	14,237 (10.0)	7008 (13.8)	9027 (7.9)	<.001
	Business & Entrepreneurs, n (%)	11,451 (8.0)	6458 (12.7)	7229 (5.4)	<.001
	Animals & Pets, n (%)	11,158 (7.8)	2131 (4.2)	9027 (9.9)	<.001
	Babies, Kids, & Family, n (%)	6265 (4.4)	1990 (3.9)	4275 (4.7)	<.001
	Volunteer & Service, n (%)	5176 (3.6)	2643 (5.2)	2533 (2.8)	<.001

^a^Given the non-Gaussian populations, we used Mann-Whitney U, chi-square, and Fisher exact tests to detect statistical differences among the three groups.

**Figure 4:**
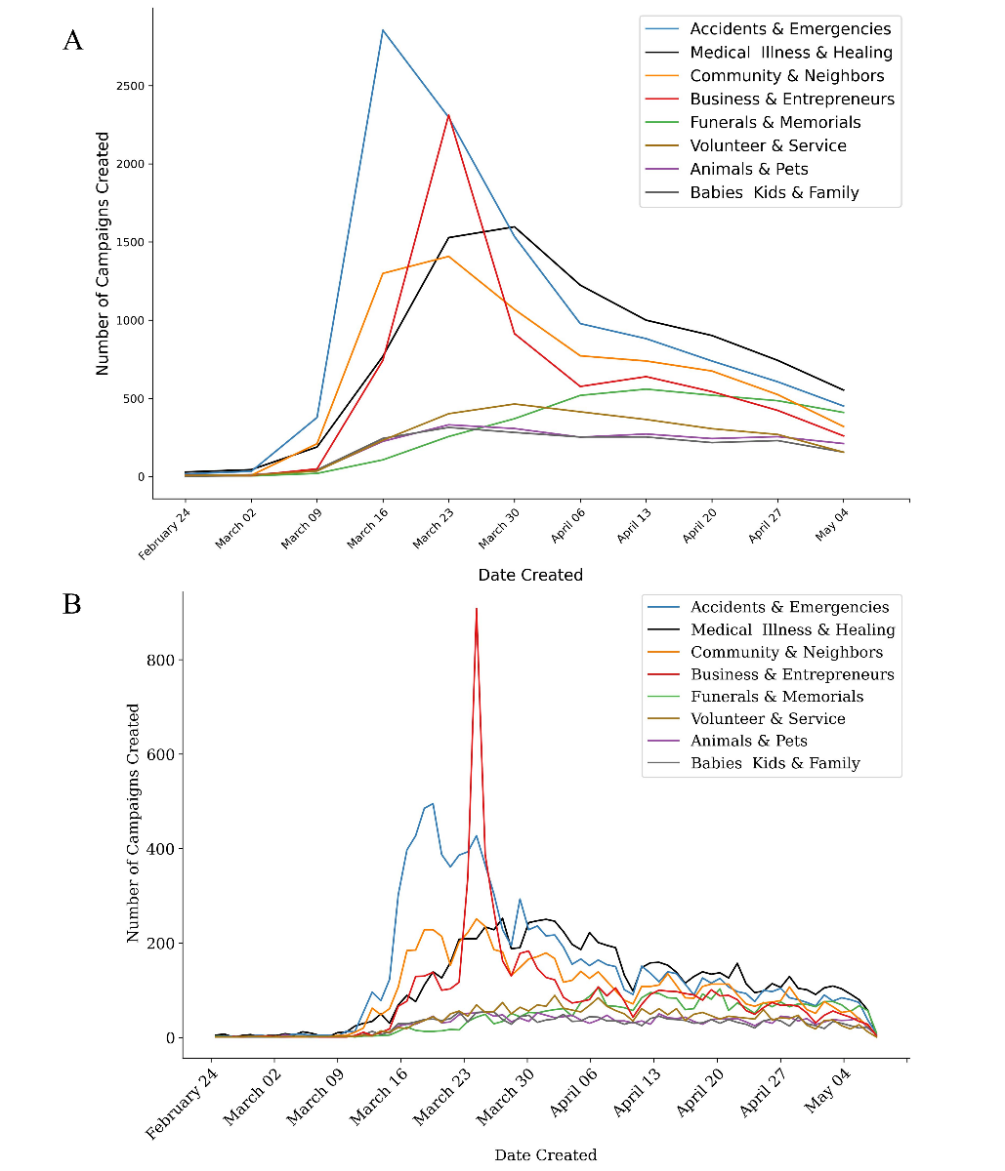
COVID-19–related GoFundMe campaigns per category (A) by week and (B) by day. Only the top 8 of the 20 possible categories are shown. The legend is ordered from most to least common category. Dates on the x-axis start from February 25, as few COVID-19–related campaigns were present before this date.

Creators of COVID-19–related campaigns most commonly selected the following categories (in descending order by number of campaigns): (1) Accidents & Emergencies, (2) Medical, Illness & Healing, (3) Community & Neighbors, (4) Business & Entrepreneurs, (5) Funerals & Memorials. In total, 19 of the 20 possible GoFundMe categories were represented in the COVID-19–related campaigns. Accidents & Emergencies as well as Community & Neighbors and Medical, Illness & Healing had the earliest peaks (Figure 4A). A week later, the category Business & Entrepreneurs peaked; however, this appeared to be driven by an isolated spike on March 24 (Figure 4B). Medical, Illness & Healing peaked next within the following 2 weeks. As these initial spikes decreased significantly, Funerals & Memorials campaigns lagged behind the first peak by 4 weeks. Compared to non-COVID-19–related campaigns after March 11, COVID-19–related campaigns had more campaigns in Accidents & Emergencies, Community & Neighbors, and Business and Entrepreneurs and fewer campaigns in Funerals & Memorials and Animals & Pets. Our NLP analysis provides granular insight into motivators for campaigns (Table 2). The largest cluster (Cluster 0) was about community support and fundraising for schools and children in a time of need. There are also multiple clusters about health care workers and personal protective equipment (Clusters 7, 11, and 18), small businesses and jobs (Clusters 3, 5, 6, and 9), as well as funerals and medical costs (Clusters 8, 15, and 17). We also observed the clustering of likely Spanish-only campaigns in Cluster 12. Cluster 19, with only 3 campaigns, likely accounts for outliers among the other campaigns and is included for completeness.

**Table 2:** Keywords at the center of the 19 clusters of campaigns obtained through the term frequency–inverse document frequency natural language processing technique and k-means clustering. The top 20 words for each cluster and the number of campaigns per cluster are displayed.

Cluster	Number of campaigns	Top 20 words in the cluster
1	10,421	community, school, support, students, time, need, pandemic, children, money, funds, thank, donation, make, continue, people, like, year, new, donations, teachers
2	6493	know, time, family, home, work, just, thank, life, need, able, baby, love, cancer, going, like, money, years, little, pandemic, people
3	4032	business, small, businesses, salon, support, open, time, community, smallbusinessrelief, clients, doors, rent, thank, able, stay, family, years, close, like, continue
4	3785	families, people, food, need, money, community, family, support, provide, supplies, time, pandemic, children, funds, feed, affected, work, basic, make, world
5	3715	job, work, pay, rent, money, just, corona, virus, car, bills, need, know, time, thank, able, family, really, lost, going, well
6	3171	staff, support, bar, time, doors, community, restaurant, family, thank, love, bartenders, know, team, members, employees, work, close, industry, times, soon
7	2935	healthcare, workers, medical, hospitals, patients, hospital, ppe, nurses, care, supplies, health, masks, equipment, protective, need, doctors, support, local, lives, working
8	2776	family, father, funeral, passed, away, life, loved, time, dad, 2020, brother, april, expenses, wife, husband, thank, know, lost, love, friend
9	2613	employees, team, restaurant, support, time, community, staff, family, doors, business, thank, customers, members, industry, restaurants, fund, work, close, crisis, open
10	2530	food, meals, local, community, restaurants, workers, support, meal, feed, provide, need, people, healthcare, families, hospital, donations, feeding, pandemic, money, responders
11	1567	masks, mask, hospitals, n95, workers, healthcare, medical, need, face, supplies, ppe, fabric, make, materials, surgical, sewing, protective, donate, hospital, purchase
12	1553	que, en, la, el, para, por, su, los, se, una, es, lo, mi, las, esta, del, familia, sus, ayuda, todos
13	1320	artists, industry, workers, fund, musicians, relief, support, community, music, service, work, people, need, funds, income, time, restaurant, financial, pandemic, assistance
14	1187	surgery, vet, dog, pain, just, life, time, know, needs, leg, family, right, old, emergency, hospital, work, going, need, cancer, tumor
15	1175	hospital, family, medical, bills, ventilator, time, blood, home, days, care, icu, prayers, thank, know, heart, life, doctors, weeks, positive, april
16	871	difference, contribution, benefit, cause, join, impact, raising, advance, thanks, making, means, donation, make, money, want, information, food, america, foundation, community
17	867	mother, family, mom, time, lost, funeral, life, thank, home, sister, daughter, know, children, son, passed, single, years, friend, work, care
18	749	shields, face, 3d, shield, ppe, printing, printer, printers, materials, masks, workers, medical, hospitals, healthcare, filament, produce, print, equipment, production, need
19	3	quarnatines, exposure, 14, response, self, support, consider, donating, days, stay, want, virus, home, family, time, alejandro, eua, eubank, eubanks, eu

## Discussion

### Principal Findings

Early in the COVID-19 pandemic, COVID-19–related campaigns were created more frequently. There was a substantial increase in overall GoFundMe web-based crowdfunding campaigns in March, mostly attributable to COVID-19–related campaigns. COVID-19–related campaigns raised more money than other campaigns, had longer narrative descriptions, and were more likely to be shared on Facebook. However, as the COVID-19 pandemic progressed, we suspect that the novelty of these campaigns wore off. As COVID-19 became a familiar and ubiquitous problem, the number of campaigns per COVID-19 case declined across all states. As proposed by Elmer et al [17], we agree that crowdfunding serves as a lens for the affective responses to the pandemic, showing both the economic uncertainties and the anxieties within a community. 

We speculate that as in commercial advertising, the creativity and novelty of a campaign as well as its quick diffusion play major roles in the campaign’s success, enabling it to progress from “friend and family funding” to crowdfunding. The subsequently declining number of COVID-19–related campaigns may be explained by potential campaign developers weighing the effort required to create a campaign with a shrinking potential for reward. Initially, the novelty and early shock of COVID-19 cases provided the chance for a campaign to “go viral”; reach many people, especially through social media; and generate large donations. Social media and mobilizing users outside the immediate family and friend network are key to attract the attention of a larger group of funders [18] and achieve crowdfunding success [19]. In fact, the use of social media has been shown to predict the volume of emergency relief donations in cases of natural disasters more accurately than conventional techniques, highlighting the importance of quick diffusion [10]. As the issue became widespread and the ability to set a campaign apart as a worthy cause became more difficult, the effort (cost) of creating a campaign may have been outweighed by the perceived likelihood of funding. As others have found, the concept of worthiness and augmenting one’s “illness narrative” become important factors in generating campaign appeal, influence, and ultimately fundraising success [6,20-22]. The states with the earliest disease burden had the fewest campaigns per case, indicating a lack of case-dependent response; this supports the premise that potential campaign designers perceive common illnesses as less likely to receive the attention of donors over time. 

We recognize that the impetus for crowdfunding is a complex interplay of many factors. Campaigns may be generated for an individual, multiple individuals, an institution such as a business, or a larger community. External factors such as government readiness, supply shortages, and lockdowns may play roles in campaign incidence. However, the rapid rise and fall in campaigns over a timeline of two or three weeks in nearly all states points to a reactionary, affective community response; it also indicates that the external factors were not meaningfully reversed or corrected during this time. The gravity and magnitude of the event likely sparked reactions of desperation, fear, and anticipation that motivated campaigners to turn reaction to action, especially with the possibility of the absence of other immediate alternatives early on.

Although web-based crowdfunding raises millions of dollars, our study suggests that it may function as a financial safety net for a limited subset of beneficiaries. First, there is ample evidence that crowdfunding success disproportionately benefits those in areas with high socioeconomic status and those with the internet and media literacy necessary to portray beneficiaries as worthy [6,21-23]. Second, medical expenses alone due to COVID-19 in the United States are estimated to be in excess of $163 billion if 20% of the population is infected (on August 26, 2020, approximately 1.8% of the population had a confirmed infection) [24]. Rather, crowdfunding more likely functions as a weathervane indicating a community in distress. As Elmer et al [17] noted, “while clearly producing an economic crisis for governments, communities, families, companies and individuals, the [COVID-19] crisis is rooted in issues of community health.” Further, crowdfunding is heavily swayed by marketing and storytelling, as it points a majority of funds toward a few cases (when these elements converge to virality) rather than aiding the many [11,25].

The large number of categories for COVID-19–related GoFundMe campaigns tells a poignant story of the broad, destructive effect that COVID-19 has had on society. Beyond creating the need to cover unexpected and costly medical expenses, the COVID-19 pandemic devastated small businesses, greatly increased unemployment, created food and housing insecurities, generated unexpected expenses such as funeral costs, and increased debt [5]. The campaign categories reflected the progression of the COVID-19 funding response, with the earliest peaks for accidents and emergencies and late growth and plateaus for funerals and memorials. Our NLP analysis provides further evidence of the content of the campaigns, as the clusters identified align with the specified categories listed.

The spike in small business campaigns was likely heavily based around the launch of the Small Business Relief Initiative on March 24, 2020 [26], and specifically, its GoFundMe.org (the charitable arm of GoFundMe) Small Business Relief Fund. The relief fund provided “one-time $500 matching grants to qualifying small businesses that created a fundraiser through the Small Business Relief Initiative or already had an existing GoFundMe until the relief fund [was] depleted.” For “fundraisers started prior to this announcement or outside of this partnership, the organizer [could] update their fundraiser description with the hashtag #SmallBusinessRelief to receive funds,” which likely accounted for the presence of this phrase in Cluster 3 in our NLP analysis. Importantly, another part of the initiative involved automatically creating fundraisers for small businesses with Yelp pages, where customers could donate directly on Yelp or on GoFundMe; however, because we only scraped GoFundMe-based campaigns that included a user-generated narrative, these fundraisers were not part of our dataset.

As newly generated GoFundMe campaigns showed a temporal association with the effect of the COVID-19 pandemic on society, we contemplated that GoFundMe campaigns may serve as an early indicator of a community in distress. To explore this, we isolated campaigns from communities that were affected by a natural disaster, including Puerto Rico (earthquake from January 6-7, 2020), Nashville (tornado from March 2-3, 2020), and Mississippi (tornado from April 12-13, 2020). We were able to discern an immediate peak for GoFundMe campaigns following each event (Figure S1, Multimedia Appendix 1).

Although GoFundMe campaigns failed to meet the needs of those seeking support, as demonstrated by the small fraction of funding goals met by the data extraction date, the signal of new campaigns at and after times of distress can provide unique insight into the community affected. The organic, user-generated content of these campaigns can inform both direct emerging needs (whether resource-related or financial) as well as the societal perception and sentiment in a time of crisis [17]. This signal does not appear to be limited to medical emergencies but also extends to natural disasters. Future research will show which types of negative events that affect communities are reflected by a spike in GoFundMe campaigns and how the content of the campaigns can be leveraged systematically.

### Limitations

Although we were able to analyze the full available set of GoFundMe campaigns, which strengthened our study, it had several limitations. Web-based crowdfunding only provides a lens to view those who have access to the technical and social resources to be a part of a crowdfunding campaign (either to create and coordinate a campaign directly or to be connected to another person who can fulfill that role). Therefore, only a subset of the population experiencing unmet financial strain or needs is represented. Moreover, we used data from one crowdfunding platform only. Although GoFundMe is the largest platform in the United States by number of campaigns, our study may have suffered from selection bias. Because of the large number of campaigns, we are unable to validate that campaigns using COVID-19–related keywords were indeed designed to mitigate the effect of COVID-19. Although we note significant differences in campaign and funding characteristics between COVID-19–related and non–COVID-19–related campaigns, future work will be important to further understand reasons for these differences and their implications in long-term crowdfunding. Finally, we only analyzed the first few months of COVID-19–related campaigns; therefore, more longitudinal analysis will be important to understand more completely how peaks and troughs in COVID-19 incidence nationally and in more granular communities affect campaign frequency.

### Conclusions

Web-based crowdfunding activity leverages galvanized public reaction early in a public health emergency such as the COVID-19 pandemic. However, as disease spread persisted and economic burden continued to grow nationally, the creation of new campaigns faded. Web-based crowdfunding appears to be a stopgap for only a minority of campaigners. The novelty of an emergency likely impacts both campaign initiation and crowdfunding success, as it reflects the affective response of a community.

More importantly than its rather weak effect of mitigating the effect of the crisis, crowdfunding activity likely provides an early signal of the emerging needs and societal sentiment of communities in acute distress. This activity could be monitored by local, state, and federal government agencies, such as the Federal Emergency Management Association (FEMA), to provided targeted support and policy. Future research will determine what emergencies generate the strongest signals in crowdfunding activities and how content can be leveraged systematically.

## Appendix

Multimedia Appendix 1 Supplementary tables and figures.

## Abbreviations

FEMA: Federal Emergency Management Association
NLP: natural language processing
TF-IDF: term frequency–inverse document frequency
